# Efficacy of transcatheter arterial embolization in treating nonvariceal gastric remnant bleeding: a retrospective 5-year study

**DOI:** 10.1186/s12876-024-03179-x

**Published:** 2024-03-04

**Authors:** Weida Wu, Jianyang Peng, Guohui Zhou, Chunsheng Zhang, Yuanzhen Lin, Zhicheng Weng

**Affiliations:** 1 School Of Clinical Medicine, Fujian Medical University, 350000 Fuzhou, China; 2 Department of Interventional and Vascular Surgery, The Affiliated Hospital of Putian University, No. 999 Dongzhen East Road, Licheng District, 351100 Putian, Fujian Province China

**Keywords:** Gastric remnant bleeding, Collateral gastric-supplying arteries, Endoscopic clips, Bleeding recurrence, Aortography

## Abstract

**Background:**

Gastric remnant bleeding is a special case of upper gastrointestinal bleeding with certain specific disease characteristics, and some matters of transcatheter arterial embolization (TAE) for hemostasis need attention. In this study, we aimed to explore the clinical use of TAE in patients with nonvariceal gastric remnant bleeding and identify the factors influencing the clinical efficacy of these interventions.

**Methods:**

Data were retrospectively analyzed from 42 patients for whom angiography and embolization were performed but could not be treated endoscopically or had failed endoscopic management in our department between January 2018 and January 2023 due to nonvariceal gastric remnant bleeding. We investigated the relationship between the incidence of re-bleeding and the following variables: sex, age, pre-embolization gastroscopy/contrast-enhanced computer tomography, embolization method, aortography performance, use of endoscopic titanium clips, and the presence of collateral gastric-supplying arteries.

**Results:**

Forty-two patients underwent 47 interventional embolizations. Of these, 16 were positive for angiographic findings, and 26 were negative. Based on arteriography results, different embolic agents were selected, and the technical success rate was 100%. The incidence of postoperative re-bleeding was 19.1% (9/47), and the overall clinical success rate was 81.0% (34/42). Logistic regression analysis of the relationship between the incidence of early re-bleeding following embolization and the proportion of collateral gastric supply arteries revealed an odds ratio of 10.000 (*p* = 0.014).

**Conclusions:**

Utilizing TAE for nonvariceal gastric remnant bleeding is safe and effective. The omission of collateral gastric-supplying arteries can lead to early re-bleeding following an intervention.

## Background

Over the past decades, transcatheter arterial embolization (TAE) has become the alternative treatment for acute upper gastrointestinal bleeding (UGIB), particularly in cases of failed endoscopic therapy. This approach has been widely used in clinical practice due to its lower invasiveness and fewer complications than those of surgery and, thus, is currently favored by clinicians [1–3]. Gastric remnant bleeding is a special case of UGIB that also presents some unique characteristics regarding interventional embolization. A remnant stomach is commonly found in conditions such as subtotal gastrectomy (Billroth I or II) performed to remove gastric lesions, including cancer or ulcers, or tubular stomach reconstruction in treating esophageal cancer. Due to the gastrointestinal tract’s postoperative anatomical and physiological changes, low gastric acid levels increase gastrointestinal reflux and bacterial overgrowth. These factors alter the microenvironment in the remnant stomach, particularly near the anastomosis.

Moreover, gastric remnants are prone to lesions, commonly including remnant gastritis, erosion, and ulcers. Notably, some lesions even become malignant, transforming into remnant gastric cancer [4]. Clinicians often need to take emergency measures to achieve hemostasis after conservative medical treatment fails to stop remnant stomach bleeding. Recent research on how to treat gastric remnant bleeding is explicitly scarce. Endoscopic therapy is the gold standard of UGIB treatment; however, bleeding is not successfully stopped in 10–25% of the patients [5], including those with gastric remnant bleeding. Therefore, surgical intervention for hemostasis should be carefully considered in patients with UGIB due to the high perioperative risk and mortality rate [6].

Furthermore, the anatomical changes of residual stomachs necessitate a careful approach when attempting emergency surgical hemostasis. In particular, reoperation for tubular stomach reconstruction after an esophageal cancer operation is a huge challenge. TAE represents a promising alternative; however, this measure also faces challenges due to the altered anatomy of the gastric remnants. This issue arises due to the disconnection between the conventional stomach-related blood vessels and the accompanying anatomical changes [7–8], often making it difficult to determine precisely which artery is responsible for the bleeding. These factors increase the difficulty of interventional embolization and affect the success rate of hemostasis. Clinicians should consider more efficient ways to locate the artery supplying the remnant stomach to improve the success rate of hemostasis. In this retrospective study, we analyzed data from patients with nonvariceal gastric remnant bleeding treated with TAE at our hospital over the past 5 years. We discuss the feasibility of utilizing TAE for gastric remnant hemorrhage treatment and explore the factors influencing re-bleeding post-embolization.

## Methods

### General information

The data of 42 patients who were treated with arteriography and embolization for acute nonvariceal gastric remnant bleeding between January 2018 and January 2023 were retrieved from the interventional therapy database of our hospital and included in the study. A gastroscopy or triphasic contrast-enhanced spiral computed tomography (CT) scan was performed before interventional embolization to confirm the presence of nonvariceal gastric remnant bleeding and initially to search for the responsible bleeding site. In this retrospective study, we collected such data to explore the efficacy of TAE in treating gastric remnant bleeding and analyze the factors leading to rebleeding. The patients’ basic information and characteristics are presented in Table 1. Informed consent for the TAE procedure was obtained from all the patients and their families, and the study was approved by our hospital’s ethics committee. Further, this study followed the ethical standards laid down in the 1964 Declaration of Helsinki and its later amendments or comparable ethical standards. The study was approved by the ethics committee of the Affiliated Hospital of Putian University of Medicine institutional review board (approval number 2,023,033).

**Table 1:** Patients and disease characteristics

Characteristics[U.]	Number(%)
Age range (Median ± SD)[years]	40–94 (67.8 ± 10.9)
Sex Male/Female	36/6
Pre-embolization gastroscopy /CT enhancement	29/13
**Underlying diseases**	
Esophageal carcinoma	13 (31.0)
Gastric carcinoma	16 (38.1)
Giant gastric ulcer	7 (16.7)
Gastroduodenal perforation	6 (14.3)
**Cause of gastric remnant bleeding**	
Gastritis	4(9.5)
Erosion	5(11.9)
Ulcer	21(50.0)
Cancer	9(21.4)
idiopathic	3(7.1)
**Blood vessels of interventional embolization**	68 (100)
Left gastric artery	24 (35.4)
Right gastric artery	7 (10.3)
Short/posterior gastric artery	6 (8.8)
Left gastroepiploic artery	2 (2.9)
Right gastroepiploic artery	14 (20.6)
Inferior phrenic artery	9 (13.2)
Intercostal artery	1 (1.5)
Internal thoracic artery	2 (2.9)
left hepatic artery	1 (1.5)
Thyrocervical trunk	2 (2.9)
**embolic agents**	
microcoils	16 (38.1)
gelatin sponge particles	34 (81.0)
gelatin sponge strips	8 (19.0)
polyvinyl alcohol (PVA) granules	8 (16.7)
surgical glue	1 (2.4)
**Therapeutic outcome**	
The number of interventional embolization	47
Technical success	42 (100)
Clinical success	35 (83.3)
Postoperative rebleeding	8 (19.0)
Surgical treatment after TAE	5 (11.9)
Mortality	5 (11.9)

CT, Computerized tomography; TAE, Transcatheter arterial embolization

The inclusion criteria were as follows: (1) repeated massive hematemesis or melena (requiring transfusion of at least four units of blood every 24 h); (2) hemodynamically instability (systolic blood pressure < 90 mmHg, heart rate > 100 beats/min, or clinical shock due to bleeding); (3) advanced age or a poor heart function and an inability to tolerate endoscopic examination and treatment; (4) at least one failed prior attempt to control the bleeding through endoscopic therapy [9]; and (5) a surgeon assessment recommending against surgical treatment for patients requesting surgical intervention. Therefore, TAE was considered if any of the above inclusion criteria were met.

The exclusion criteria comprised the following: diagnosis with variceal gastrointestinal bleeding, TAE refusal for any reason, or a request for surgical treatment.

### Interventional procedures

(1) All patients were routinely prepped for venous access before the interventions. Fluid volumes were actively replenished, coagulation disorders were treated, and blood transfusions were performed where appropriate. (2) The femoral artery sheath was locally anesthetized with 2% lidocaine, and the modified Seldinger technique was used to puncture the femoral artery. A 5 F sheath was inserted through the femoral artery. Furthermore, various angiography catheters were employed for arterial angiography, followed by super-selective arteriography using 2.7 F microcatheters (Terumo Corporation, Maimaigi - Cho Fujinomiya City, Japan). (3) For patients with positive arteriography results, different types and doses of embolic agents were selected to perform therapeutic embolization after the bleeding artery was identified using the angiography results. These embolic agents included micro coils (Boston Scientific Corporation, MA, USA), surgical glue (GLUBRAN2, GEM S.R.L. Gemm LTD., Italy), gelatin sponge particles (Hangzhou Alicon Pharm SCI & TEC CO., LTD., Hangzhou, China), gelatin sponge strips, and polyvinyl alcohol (PVA) granules (Cook Inc., IN, USA). (4) For patients with negative arteriography results, empiric embolization was performed based on the bleeding site’s location, confirmed through contrast-enhanced CT, endoscopy, or by referencing the titanium clip location. In the absence of endoscopy, if contrast-enhanced CT also showed no positive findings, decompression embolization was performed to embolize all potential supplying arteries of the gastric remnant. Embolic agents included gelatin sponge particles, strips, and PVA particles. (5) Positive arteriography results included direct signs of contrast agent extravasation and indirect signs such as pseudoaneurysm, submucosal vascular malformation, and intramucosal contrast agent deposition (observed in cases with tumors) [10]. Negative arteriography results were defined as the absence of the signs mentioned above.

### Evaluation criteria and postoperative follow-up

(1) Failed endoscopic management was defined as follows: (a) active bleeding precluded the observation of the endoscopic field during endoscopic treatment or resulted in unstable vital signs, making the patient unable to complete endoscopic therapy; (b) inability to undergo endoscopic treatment: endoscopy was highly suggestive of gastric remnant cancer or serious gastric lesions such as multifocal ulcers, extensive erosion, or giant ulcers which could not be managed using endoscopic hemostasis. (2) Embolization methods included prophylactic (empiric and decompression embolisms) and therapeutic embolizations. The difference in embolization methods was based on the control of the endpoint. The endpoint of the therapeutic or empiric embolization was the disappearance of the responsible bleeding artery as observed on a repeated angiography. The endpoint of decompression embolization was a slow blood flow and a distal end block for the potential arteries, as observed on imaging with repeated angiography. (3) The feeding arteries of the stomach were defined as conventional gastric supply arteries, such as the left/right gastric artery, the left/right gastroepiploic artery, and the short (posterior) gastric artery. The collateral gastric supply arteries were gastric supply arteries other than the arteries mentioned above. (4) Technical success was defined as completion of the planned angiography and embolization. Clinical success was defined as the absence of re-bleeding after embolization. (5) Postoperative re-bleeding mainly manifested as a recurrence of hematemesis and bloody stools and decreased hemochrome or unstable vital signs. Early recurrence of bleeding (or “re-bleeding”) was defined as that occurring within 7 days postoperatively. Mid-term recurrence of bleeding was defined as that occurring 7–30 days postoperatively, and late recurrence was defined as that occurring > 30 days postoperatively.

### Statistical analysis

We used SPSS Statistics version 19.0 (IBM Corp., Armonk, NY, USA) to analyze the data. All normally distributed measurement data are presented as means ± standard deviations (x ± s). Count data are expressed as case numbers or rates. Binary chi-square and independent-sample T-tests of the factors causing early re-bleeding were used for single-factor analysis. The related indicators including age, sex, embolization method, aortography, pre-embolization gastroscopy/CT enhancement, use of endoscopic titanium clips before intervention, and the existence of collateral gastric supplying arteries in a single-factor analysis were brought into single-factor logistic regression analysis respectively. Two-sided tests were used, and statistical significance was set at *p* < 0.05.

## Results

Forty-two patients with nonvariceal remnant gastric bleeding were included in this study, all of whom completed TAE. There were 36 men and six women aged 40–94 (mean: 67.8 ± 10.9) years. There were 13 cases of residual gastric hemorrhage following esophagectomy and 29 cases of partial gastrectomy. The endoscopic manifestations of gastric remnant bleeding (Table 1) included four cases (9.5%) of gastritis, five (11.9%) of erosions, 21 (50.0%) of ulcers, and nine (21.4%) of cancer. Three patients (7.1%) with unknown diagnoses were unable to undergo endoscopy due to poor cardiopulmonary function and advanced age.

### Results of transcatheter arterial embolization

Notably, all 42 patients underwent interventional embolization therapy, with 47 procedures performed. One patient underwent three interventional therapies for re-bleeding, three underwent two interventional therapies for re-bleeding, and 38 underwent one. The technical success rate of the embolization therapy was 100%. Sixteen cases were positive, including nine with direct signs and seven with indirect signs, of which two had pseudoaneurysms, four had submucosal vascular malformations, and one had tumor contrast staining. The gastric supply arteries were embolized as follows (Table 1): the conventional gastric supply arteries included 24 cases (35.4%) where the left gastric artery was treated, seven (10.3%) involved the short/posterior gastric artery, seven (10.3%) involved the right gastric artery, two (2.9%) involved the left gastroepiploic artery, and 14 (20.6%) involved the right gastroepiploic artery. The collateral gastric supply arteries included nine cases (13.2%) that involved the inferior phrenic artery, one (1.5%) that involved the intercostal artery, two (2.9%) that involved the internal thoracic artery, and two (2.9%) where the thyrocervical trunk was treated. There were 16 cases of therapeutic embolism, 10 of empirical embolism, and 16 of decompressive embolism. The decision for use of embolization agents during TAE was based on a combination of the cause of gastric remnant bleeding and the lesions of the responsible vessels with the evaluation of contrast-enhanced CT and angiography, specifically as follows (Table 1): coil embolization in two cases, PVA particles in six, gelatin sponge particles in 20, coils combined with gelatin sponge particles in 12, PVA particles combined with gelatin sponge particles in one, and one case where coils and gelatin sponge particles were both combined with surgical glue.

### Effects of interventional therapy, complications, and postoperative follow-up

The overall clinical success rate was 83.3% (35/42), of which the rate for therapeutic embolization was 93.8% (15/16) and that for prophylactic embolism was 76.9% (20/26). There was no significant difference between the two groups of embolization methods regarding the incidence of postoperative recurrence of bleeding (χ^2^ = 0.989; *p* = 0.320). The mortality rate was 11.9% (5/42) as a result of serious underlying diseases, massive hemorrhages, multiple organ failures, and other causes. The five patients (11.9%) with gastric remnant cancer after subtotal gastrectomy underwent surgical resection within a defined time following embolization, and re-bleeding did not occur in any of these cases over the follow-up period. The rate of re-bleeding during follow-up was 19.0% (8/42), of which the early rate was 14.3% (6/42), the mid-term rate was 2.4% (1/42), and the early and late simultaneous re-bleeding rate was 2.4% (1/42). Of all patients, 28.6% (12/42) were found with the collateral gastric-supplying artery during the intervention procedures, and there was a significant impact on the rate of re-bleeding for this group compared with the group without these arteries (χ^2^ = 5.25; *p* = 0.022, Table 2). Simultaneous logistic regression analysis (Table 3) showed that the group with these arteries positively correlated with early re-bleeding (OR = 10.000; *p* = 0.014). The participation of the collateral gastric supply arteries may result in the absence of parts of the responsible artery, leading to early postoperative re-bleeding.

**Table 2:** Analysis of the potential factors affecting early re-bleeding

Factor	Case	Re-bleeding (*n* = 42)	χ^2^/t	P value
Age	42	7 (16.7)	0.276	0.784
**Sex**				
Male	36	6 (16.7)	<0.001	1
Female	6	1 (16.7)		
**Embolization method**				
Prophylactic embolization	26	6 (23.1)	0.989	0.32
Therapeutic embolization	16	1 (6.3)		
**Aortography**				
Yes	12	1 (8.3)	0.21	0.647
No	30	6 (20.0)		
**Pre-embolization gastroscopy /CT enhancement**				
Gastroscopy	29	6 (20.7)	0.356	0.55
CT enhancement	13	1 (7.7)		
**Use of endoscopic titanium clips before intervention**				
Yes	7	2 (28.6)	0.137	0.711
No	35	5 (14.3)		
**The existence of collateral gastric supplying arteries**				
Yes	12	5 (41.7)	**5.25**	**0.022**
No	30	2 (6.7)		

Bold text indicates significant differences with *p* < 0.05

**Table 3:** Single-factor logistic regression analysis of the related factor affecting early re-bleeding

Factor	β	SE	Wald /χ2	P value	OR	95% CI
Age	−0.011	0.039	0.079	0.778	0.989	0.917–1.067
Sex	<0.001	1.183	<0.001	1.000	1.000	0.098–10.166
Embolization method	−1.504	1.133	1.763	0.184	0.222	0.024–2.047
Aortography	−1.012	1.140	0.788	0.375	0.364	0.039–3.395
Pre-embolization gastroscopy /CT enhancement	−1.141	1.137	1.007	0.316	0.319	0.034–2.968
Use of endoscopic titanium clips before intervention	0.875	0.966	0.821	0.365	2.400	0.361–15.942
Existence of collateral gastric supplying arteries	2.303	0.937	6.035	**0.014**	**10**	1.593–62.784

Bold text indicates statistically significant differences with p values < 0.05. SE, Standard Error; OR, Odds Ratio; CI, Confidence Interval

After embolization, all patients were continually treated with fasting, drug hemostasis, total parenteral nutrition, and blood transfusions, if necessary. Gastroscopy was performed to confirm whether bleeding had been stopped, and 39 patients underwent follow-up endoscopy after TAE to confirm only slight ischemic injury of the gastric mucosa within 1 week. No serious complications, such as necrosis, perforation, or ectopic embolism, occurred in any of the patients during or after their procedures. Common complications such as thoracalgia (14.3%, 6/42), abdominal pain (21.4%, 9/42), low fever (21.4%, 9/42), nausea (23.8%, 10/42), and vomiting (9.5%, 4/42) were relieved within 3–5 days of pharmaceutical treatments.

## Discussion

**Fig. 1:**
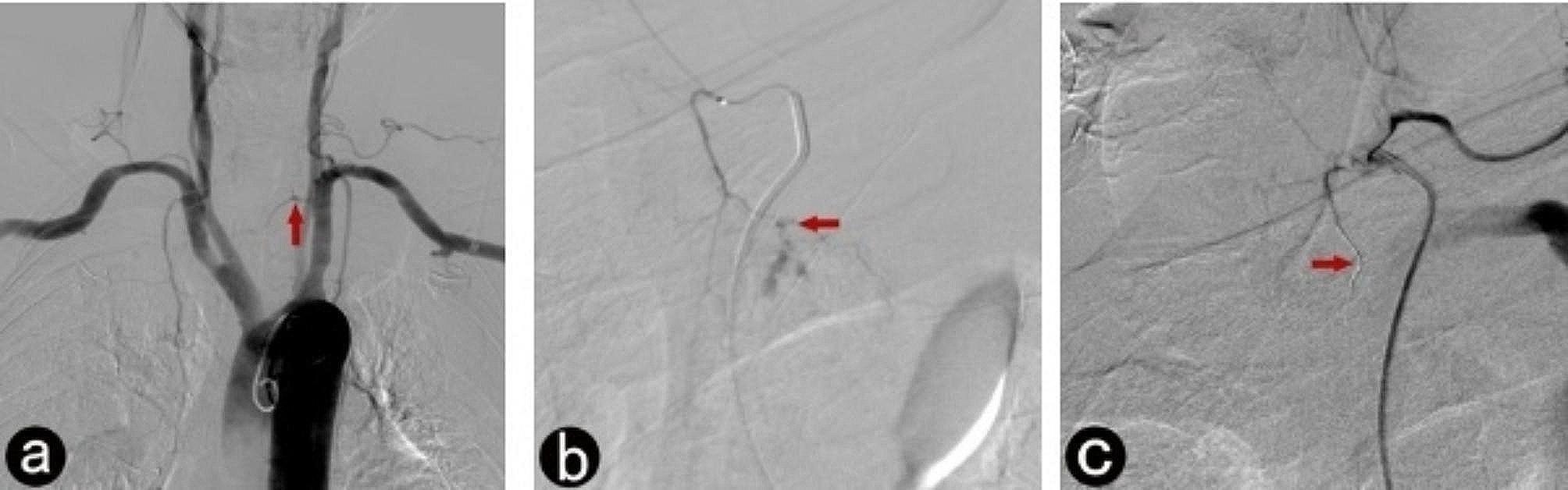
Interventional therapy for esophago-gastric anastomotic bleeding, tubular stomach reconstruction in a 74-year-old man. **a**, Aortic arch angiography reveals slight contrast agent spillage from a branch of the left thyrocervical trunk (arrow); **b**, Subsequent angiography confirms the bleeding artery responsible for the hemorrhage (arrow) after microcatheter superselection; **c**, Blocked blood flow can be seen following a micro coil embolization (arrow)

**Fig. 2:**
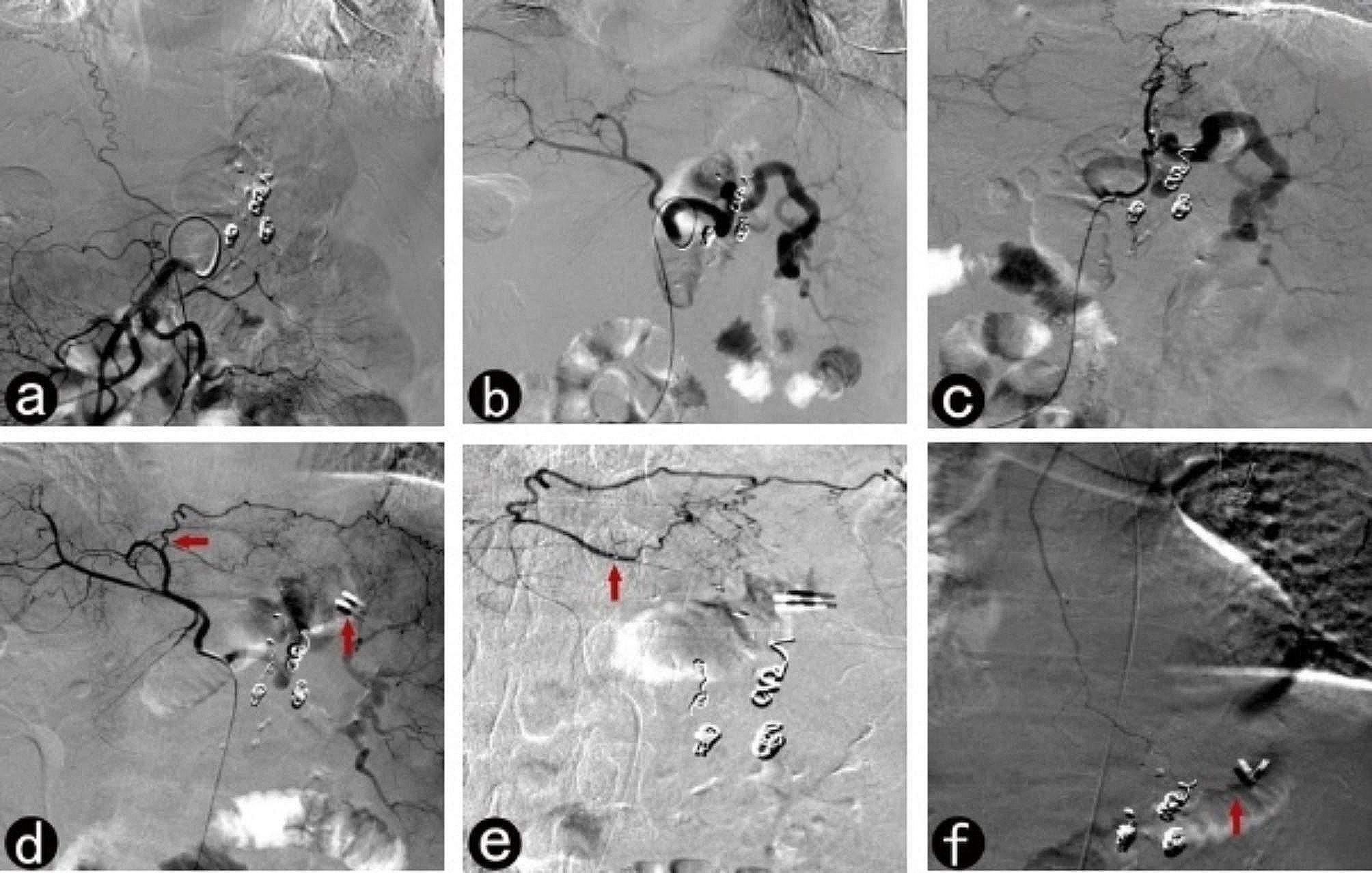
Interventional therapy for gastric remnant hemorrhage post-Billroth II subtotal gastrectomy in an 85-year-old man. a, b, No stomach-related blood supply arteries are seen on angiographies of the celiac axis and superior mesenteric arteries, respectively; c, A microcatheter is superselected to the left phrenic artery, after which gelatin sponge particles are used for decompressive embolization; d, Initial re-bleeding persisted despite using an endoscopically-implanted titanium clamp. A repeat angiography reveals a branch of the left hepatic artery (arrow) flow towards the titanium clips (arrow); e, A microcatheter is superselected to the suspected bleeding vessel, followed by empiric embolization using gelatin sponge particles; f, The terminal branch of the left internal thoracic artery is found to run near the titanium clips (arrow), and embolization is performed similarly

**Fig. 3:**
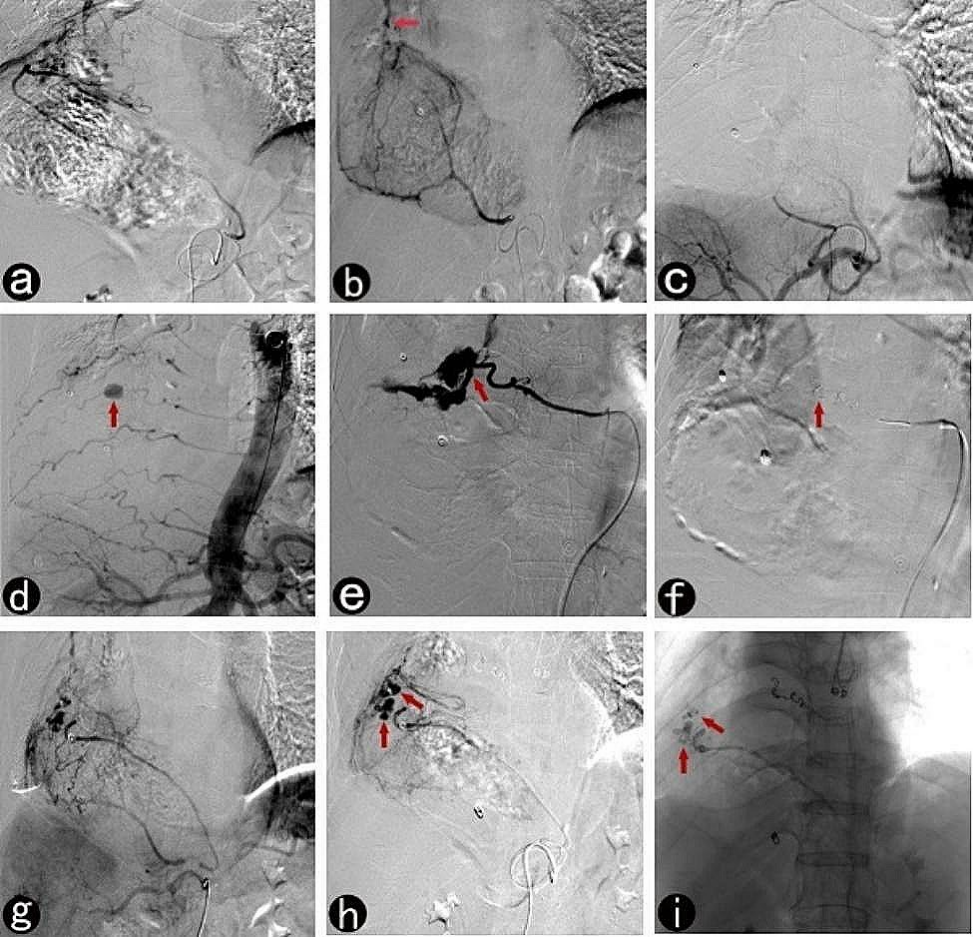
Interventional therapy for gastric remnant bleeding and tubular stomach reconstruction in a 65-year-old man. **a**, Arteriography with microcatheter shows that the distal end of the right gastroepiploic artery contains disorganized and malformed vessels; **b**, After the right gastroepiploic artery embolized, arteriography with microcatheter shows arteriovenous malformation (the early draining vein, arrow) at the distal end of the right gastric artery; **c** A repeat angiography with an angiography catheter performed 4 days postoperatively to investigate the cause of re-bleeding indicates persistent blockage of blood flow in the right gastric and right gastroepiploic arteries due to the use of micro coils combined with gelatin sponge particles; **d**, Aortography with angiography catheter shows a pseudoaneurysm (arrow) at the distal end of the intercostal artery; **e**, A microcatheter is inserted further into the responsible intercostal artery to confirm the location of the bleeding (arrow); **f**, Repeat angiography following embolization using micro coils (arrow) shows blockage of blood flow; **g**, After 39 days postoperatively, a celiac arteriography with angiography catheter shows that the blood flow in the right gastric and right gastroepiploic arteries reopened, leading to re-bleeding; **h**, A super-selective arteriography of the right gastroepiploic artery, done using a microcatheter, shows signs of multiple intracranial aneurysm (arrow) in the distal portion; **i**, A surgical glue-lipiodol (1:2) emulsion is used to embolize the responsible vessel (arrow) until the blood flow is fully blocked

Gastric remnant bleeding is a type of UGIB. The remnant stomach can ligate part of the original stomach-supplying arteries and change its original position in various ways based on the surgical method used, leading to some collateral gastric-supplying arteries potentially supplying blood to the remnant stomach. These collateral arteries may include the intercostal, thyrocervical trunk, internal thoracic, inferior phrenic, and other arteries. In this retrospective study, 28.6% (12/42) of patients were found to have collateral gastric-supplying arteries, whereas five in seven patients with early re-bleeding were found to have such arteries. This indicated that patients with such arteries were more prone to early re-bleeding. Treating such arteries responsible for bleeding is a major challenge for clinicians. If these arteries are missed, they can cause interventional therapy failure. Another great challenge of TAE was that some patients with negative angiography suffered from bleeding from collateral gastric-supplying arteries. In this study, decompression or empirical embolization was adopted, and good efficacy was achieved, with a success rate of 76.9%. However, the main problem was the anatomical change of the residual stomachs, which made it difficult to determine whether the collateral arteries participated in their blood supply. Accordingly, finding other collateral arteries that cause bleeding is critical. In this study, 29/42 patients had undergone endoscopy. Among these, hemorrhagic foci could not be observed in some patients due to massive bleeding. Additionally, bleeding could not be stopped under endoscopy in some patients due to the wide range of serious residual gastric lesions. Titanium clips should be routinely used at this point, which could be conducive to helping identify collateral arteries (Fig. 2, d-f). Aortography could sometimes help discover collateral arteries, especially when patients have positive signs of contrast agent extravasation (Fig. 1). The absence of preoperative contrast-enhanced CT or computed tomography angiography (CTA) to assist in vascular assessment may lead to the omission of the intercostal artery (Fig. 3, d), eventually leading to re-bleeding. Moreover, CTA is useful for evaluating postoperative anatomical changes and bleeding vessels [11]. In summary, endoscopic titanium clips, intraoperative aortography, and preoperative contrast-enhanced CT or CTA can be performed to identify the responsible bleeding vessel to a certain extent [12–14].

Generally, gastrointestinal bleeding caused by vascular malformation is the most serious type, as shown in Fig. 3, a,b,g,h. Its etiology and pathogenesis remain unclear, although it may be associated with acquired vascular degeneration and mucosal degeneration associated with chronic ischemia following gastric surgery [15]. Bleeding caused by residual gastric tumors is second only to vascular malformation bleeding, and endoscopic treatment often has a poor effect on this type of bleeding [16]. It is essential to quickly and accurately perform interventional embolization to stop bleeding in these cases. Notably, some patients who suffer from these phenomena are unable to undergo endoscopic examinations due to their heavy bleeding. Therefore, interventional embolization may need to be used as a preferred alternative emergency diagnostic method, making it a vital part of rescuing such patients [17]. In general, the postoperative effect of embolization therapy on patients with gastric remnant cancer is often unpredictable because the cause of the re-bleeding may be associated with tumor progression. These patients should be assessed for their ability to tolerate surgery, as surgical resection is recommended within a limited time to prevent the recurrence of gastrointestinal bleeding. Interventional embolization should be prioritized in patients who have recovered physically before surgical resection to reduce perioperative risk and mortality. In this study, nine patients had gastric remnant cancer and hemorrhage, of whom five underwent surgical resection within a week following their initial interventions, recovered well, and were subsequently discharged from the hospital. One patient discontinued treatment and ultimately died, and the remaining three underwent chemotherapy for oncological control after hemostasis was achieved.

Deciding which embolic agents should be used for interventional therapy to treat gastrointestinal bleeding remains controversial [1]. Bleeding in different parts of the digestive tract and the underlying causes vary, resulting in different embolization techniques, microcatheters, and embolic agents used for different cases [18]. As for the selection of embolic agents, our center’s experience is that permanent embolic agents such as micro coils, surgical glue, and PVA granules are prioritized for therapeutic embolism. In contrast, medium-term embolic agents such as gelatin sponge particles and strips are prioritized for prophylactic embolism. In this study, all patients with prophylactic embolism were treated with medium-term embolic agents, except PVA granules, used for residual gastric tumors. Notably, some patients could not undergo gastroscopies for various reasons before their interventions and/or showed no positive signs on angiography and contrast-enhanced CT. Decompression embolization was adopted for these patients. However, prophylactic embolism remains controversial in clinical practice [10, 19]. At this time, titanium clip treatment, used as a marker for empiric embolization, can partly help improve the success rate of prophylactic embolization [10], as shown in Fig. 2.

There were eight patients with recurrent hemorrhages following interventional therapy, six of whom had early re-bleeding after prophylactic embolization. This early re-bleeding may be associated with the omission of the responsible gastric supplying arteries. One case had mid-term re-bleeding after 2 weeks, the cause of which was considered to be a possible residual gastric tumor based on their increased carcinoma embryonic antigen levels. This patient’s family declined treatment. One patient experienced re-bleeding twice, along with early (Fig. 3, c-f) and late recurrences (Fig. 3, g-i). The cause of the early recurrence was the omission of the intercostal artery during the first intervention. The cause of the late recurrence was the improper use of embolic agents. The patient was treated with therapeutic embolization using micro coils combined with gelatin sponge particles to treat gastric vascular malformation. After 39 days, the responsible arteries reopened, and bleeding recurred due to the absorption of the gelatin sponge particles and micro coils that were too small and short to block the responsible arteries. Surgical glue embolization was then selected to stop the bleeding with a second procedure, and no further recurrence was observed after the patient’s discharge. Embolization materials should be selected based on residual gastric characteristics and angiography results. Permanent embolization agents should be used in patients with therapeutic embolization to reduce the possibility of re-bleeding. In cases where the scope of the vascular lesions is too large or the path for reaching the target vascular lesions is too long, the microcatheter cannot be superselectively accessed to target vessels for microcoil embolization. At this time, it should be noted that permanent peripheral embolization agents such as surgical glues and PVA particles should be given priority in the case of vascular malformations; however, the use of such embolization agents should be done carefully to avoid microcatheter blockage.

No serious complications, such as gastrointestinal necrosis or perforation, occurred in this study’s patients. Thirty-nine patients underwent follow-up endoscopy after TAE to confirm the absence of severe slight ischemic injury of the gastric mucosa within 1 week after the procedure. Another three patients were unable to undergo endoscopy due to poor cardiopulmonary function and advanced age, and no evidence of residual gastric necrosis or perforation was found during follow-up within 1 month after embolization. However, clinicians in this field should remain aware of the possible occurrence of gastrointestinal ischemic necrosis caused by insufficient residual stomach blood supply following interventional embolization. This is because some conventional gastric supply arteries are typically cut off during subtotal gastrectomy or esophageal cancer surgeries. In this study, embolization therapy was found to be safe for remnant gastric bleeding over a follow-up period of 0.5–5 years following subtotal gastrectomy and 11 months to 10 years following esophageal cancer surgery. This may be because other collateral gastric arteries can supply the residual stomach. However, more cases are required to confirm the safety of interventional embolization, particularly for patients with tubular stomach reconstruction bleeding following esophageal cancer surgery. If remnant stomach hemorrhage occurs within a short time postoperatively, collateral branch compensation of the residual stomach may not be established. Therefore, clinicians still need to pay attention to whether the risk of anastomotic ischemic necrosis or perforation may occur following an interventional embolization.

This study has some limitations. First, this was a retrospective and single-center study with few patients. Due to the small sample size of this study, no more independent variables could be included for logistic regression, and only single-factor logistic regression was carried out. Statistical methods could not be used to control confounding bias among the variables, which may have an impact on the conclusion. Thus, the conclusion of this study is only a preliminary exploration. The sample size should be increased in future studies and the follow-up time should be extended, to obtain more meaningful conclusions. Second, the mid and long-term curative effects of hemorrhages caused by malignant residual stomach lesions could not be evaluated in this study because most patients were further controlled with combined therapies such as surgical operations or chemotherapy after embolization to obtain a longer-term hemostatic effect. Third, 11.9% (5/42) of patients died for various reasons, affecting the observation of re-bleeding in the mid and long-term follow-up periods.

## Conclusions

As there were different types of embolic agents used in the same patient in the study, it was impossible to determine whether re-bleeding was associated with the type of embolic agent. Additionally, the postoperative follow-up period was not long enough to estimate the long-term complications of using TAE to control remnant gastric bleeding, including the stenosis of the site of the anastomosis. A multicenter joint case-control study should be set up to further validate our research results and draw additional conclusions.

Different embolization agents and methods can be used to treat residual stomach hemorrhage based on the results of gastroscopy, contrast-enhanced CT, or arteriography before embolization. The early re-bleeding following interventional therapy may be associated with missed collateral gastric-supplying arteries during initial interventions. Preoperative gastroscopy, contrast-enhanced CT, endoscopic titanium clips, or intraoperative aortography may help detect these potential collateral gastric feeding arteries, increasing the clinical success rate of interventional procedures.

## Data Availability

The data presented in this study are available on request from the corresponding author.

## Abbreviations

CT: computed tomography
CTA: computed tomography angiography
TAE: transcatheter arterial embolization
PVA: polyvinyl alcohol
UGIB: upper gastrointestinal bleeding
